# Hospital Admission following Acute Kidney Injury in Kidney Transplant Recipients Is Associated with a Negative Impact on Graft Function after 1-Year

**DOI:** 10.1371/journal.pone.0138944

**Published:** 2015-09-29

**Authors:** Thiago Corsi Filiponi, Lúcio Roberto Requião-Moura, Eduardo José Tonato, Ana Cristina Carvalho de Matos, Alvaro Pacheco e Silva-Filho, Marcelino de Souza Durão Junior

**Affiliations:** 1 Kidney Transplant Unit, Hospital Israelita Albert Einstein, São Paulo, Brazil; 2 Nephrology Division, Universidade Federal de São Paulo, São Paulo, Brazil; University of Florence, ITALY

## Abstract

The incidence and outcomes of acute kidney injury (AKI) in kidney transplantation are poorly known. Retrospective cohort analysis was performed on the data of all patients (≥3 months after transplantation and ≥16 years of age) admitted to the hospital due to medical or surgical complications from 2007 to 2010. We analyzed 458 kidney transplant recipients, 55.2% men, median age 49 (IQR, 36–58) years, median of 12.5 (IQR, 3–35) months after kidney transplantation; admitted to the hospital due to medical or surgical complications. Most of the patients received a kidney from a deceased donor (62.2%), the primary cause for hospital admission was infection (60.7%) and 57 (12.4%) individuals were diagnosed with acute rejection (AR). The incidence of AKI was 82.3%: 31.9% stage 1, 29.3% stage 2 and 21.2% stage 3. Intensive care unit (ICU) admission (OR 8.90, 95% CI: 1.77–44.56 p = 0.008), infection (OR 5.73, 95% CI: 2.61–12.56, p<0.001) and the use of contrast media (OR 9.34, 95% CI: 2.04–42.70, p = 0.004) were the independent risk factors for AKI development. The mortality rate was 2.1% and all patients who died were diagnosed with AKI. Even after the exclusion of AR cases, at the end of 12 months, the individuals with AKI exhibited higher percent changes in creatinine values when compared with individuals without AKI (9.1% vs. -4.3%; p<0.001). According to KDIGO system, we found a high incidence of AKI among the complications of renal transplantation. As in other scenarios, AKI was associated with renal function loss at 1-year after the hospital discharge.

## Introduction

Recent data reveal that AKI is an independent risk factor for mortality in the short and long term, and its occurrence increases the treatment complexity, length of hospital stay, and treatment costs [1]. AKI is also a risk factor for the development of chronic kidney disease (CKD) and dialysis dependence [2].

Renal transplant recipients are susceptible to developing AKI because they have a decreased renal ‘reserve’ due to reduced mass of functioning nephrons. The ischemia-reperfusion injury developed during the kidney transplant procedure, the use of nephrotoxic drugs, especially calcineurin inhibitors, and immune-mediated injury are also risk factors for renal graft impairment [3].

Data on AKI development in renal transplant recipients and its impact on graft function are scarce in the literature. Thus, we decided to study the incidence and outcomes of AKI in this particular group using the KDIGO (Kidney Disease: Improving Global Outcomes) criteria [4]. We evaluated need for dialysis, mortality, and renal function during 1 year after hospital discharge.

## Subjects and Methods

This retrospective cohort study assessed the incidence of AKI in renal transplant recipients admitted to the Hospital Israelita Albert Einstein, Sao Paulo-Brazil, due complications between February-1-2007, and December-31-2010.

Demographic, clinical, and laboratory data were collected from the medical records.

The study was approved by the local ethics committee (number-1335-11). Informed consent was waived because of the nature of the study.

### Inclusion criteria

All renal transplant recipients≥16 years old with time since transplantation ≥ three months were considered for this study. Only patients with a single admission during the study were considered.

### Exclusion criteria

Individuals with less than 48 hours (minimum time required for AKI) of hospital stay, patients with multiple admissions (multiple injuries), and subjects who underwent transplantation of the kidney combined with another organ were excluded. Recipients with post-renal AKI were excluded as well.

### Follow-up time

All patients were followed for 1 year after hospital discharge.

### Variables

The following recipient variables were assessed: age; gender; race; CKD etiology; and presence of comorbidities, such as diabetes, arterial hypertension, and cardiovascular disease (CVD) at admission, according to previously established definitions [5–8].

The post-transplant variables analyzed were type of donor, time between the transplantation and hospital admission in months, use of calcineurin inhibitor, and whether the patient was induced with an antilymphocyte globulin.

### Baseline renal function

The baseline renal function was obtained using the mean value of the last three stable levels of plasma creatinine, measured at the outpatient clinic before the event that led to hospital admission. Baseline glomerular filtration rate was estimated (eGFR) by the abbreviated MDRD equation. Creatinine was measured monthly from three to twelve months after the transplant, every two months from the first to the second year after the transplant and every three months from the second year onward. During hospital stay, serum creatinine was measured daily; all individuals with kidney function impairment underwent routine Doppler ultrasound of the renal graft.

### Hospital admission causes

Causes of hospital admission were classified as infectious; cardiovascular; due to undetermined graft dysfunction (see below), tumors, or surgeries; or other. The infections were classified as bacterial, viral by cytomegalovirus (CMV) or other infections.

### Graft biopsy and Acute Rejection (AR)

Changes in renal function without an evident triggering factor (e.g. infection, drugs nephrotoxicity, hypotension, etc.) were considered undetermined graft dysfunction. These patients were usually admitted with a suspicion of rejection and an indication for renal graft biopsy. We observed cellular and antibody-mediated rejection as defined by the Banff criteria [9] and according to the presence of other histological findings.

### Nephrotoxic drugs

During the hospital stay, we assessed the use of nephrotoxic drugs (vancomycin, amphotericin B, iodinated contrast media, polymyxin B, aminoglycosides, and chemotherapy agents, such as, cisplatin, carboplatin and etc.).

Patients who underwent contrast examination routinely received 0.9% saline solution 1 ml/kg/hour, 24 hours before and 24 hours after investigation, and those who needed to undergo the procedure as an emergency received 1 liter of 0.9% saline solution before and after the contrast media use. Serum vancomycin levels were measured in patients to keep the levels between 10 and 20 ng/ml (valley).

Routinely, adjustments dose of tacrolimus and cyclosporine were made to maintain target throughlevels between 5–8 ng/ml and 100–150 ng/ml, respectively. During an AKI episode, calcineurin inhibitors were not withdrawn. In the presence of severe sepsis or septic shock immunosuppression was minimized and the patients were kept only with corticosteroids.

### Intensive Care Unit

We evaluated the need for and length of stay in ICU, the use of mechanical ventilation, vasopressors, and sedation.

### AKI diagnosis and classification staging

AKI diagnosis and classification were established by the KDIGO criteria and were based only on the change in plasma creatinine (S1 Table). The urine output was not used as a criterion because most of patients had no indwelling bladder catheter, hindering reliable analysis of urine volume.

### Outcomes

With regard to the outcomes, we assessed the renal function (plasma creatinine) over twelve months after hospital discharge. We also assessed the need for acute (in-hospital) and chronic (dependence at hospital discharge) dialysis and mortality during the study.

### Statistical analysis

The categorical variables were reported as absolute frequencies and percentages. The distribution of the numerical variables was assessed using the Shapiro-Wilk test, and the variables were reported as medians and interquartile ranges (IQR, 25-75th).

The analysis of the factors associated with AKI development, requirement for dialysis, and death was performed using Pearson’s chi-square test or Fisher’s exact test for categorical variables and the Mann-Whitney non-parametric test for numerical variables. Variables with p-values less than or equal to 0.10 were included in the multiple logistic regression models. The models were subjected to stepwise variable selection, and only the variables with p-values less than 0.05 were kept in the final model.

The creatinine levels in patients with and without AKI during the study were analyzed using Friedman’s non-parametric test, and the groups were compared using the Mann-Whitney non-parametric test.

The analyses were performed using the software SPSS (Version-17.0; Chicago-SPSS, Inc.), and the significance level adopted for all tests were 5%.

## Results

### Patient characteristics and clinical data

Four hundred and fifty eight individuals met the inclusion criteria. The main clinical and demographic data are described in Table 1.

**Table 1:** Demographic and clinical data.

Variables	no-AKI (N = 81)	AKI(N = 377)	P value
Age–years(IQR)	46 (40–58)	49 (36–58)	0.860^#^
Male (%)	41 (50.6)	212 (56.2)	0.356^¶^
Caucasian (%)	50 (61,7)	226 (59.9)	0.591*
Comorbidities (%)			
Diabetes Mellitus	30 (37.0)	134 (35.5)	0.799^¶^
Hypertension	56 (69.1)	285 (75.6)	0.226^¶^
Deceased donor (%)	47 (58.0)	238 (63.1)	0.390^¶^
Admission causes (%)			
Infection	40 (49.3)	238 (63.1)	<0.001*
CVD	6 (7.4)	26 (6.8)	0.872^¶^
Neoplasia	9 (11.1)	12 (3.1)	0.005*
Baseline creatinine—mg/dl	1.2 (1.1–1.6)	1.4 (1.2–1.8)	0.007^#^
Baseline eGFR—ml/min/1.73m^2^	65.0 (53.0–79.0)	62.0 (50.0–73.0)	0.082^#^
Transplant time–months	23 (6–34)	11 (4–35)	0.051^#^
Calcineurin inhibitor (%)	70 (86.4)	346 (91.7)	0.130^¶^
Contrast media (%)	2 (2.4)	74 (19.6)	<0.001^¶^
Vancomycin (%)	2 (2.4)	53 (14.0)	0.004^¶^
Amphotericin B (%)	1 (1.2)	22 (5.8)	0.097*
Chemotherapy (%)	4 (4.9)	12 (3.1)	0.501*
ICU admission (%)	2 (2.4)	81 (21.4)	<0.001^¶^
Vasopressor (%)	0 (0.0)	53 (14.0)	<0.001^¶^
Mechanical ventilation (%)	0 (0.0)	38 (10.0)	0.003^¶^
Sedation (%)	0 (0.0)	34 (9.0)	0.005^¶^

Median (IQR, 25-75th); AKI: acute kidney injury, CKD: chronic kidney disease, CVD: cardiovascular disease, eGFR:estimated glomerular filtration rate, ACEI/ARB: angiotensin-converting-enzyme-inhibitors/angiotensin receptor; blockers, ICU: intensive care unit

#: non-parametric Mann-Whitney test

¶: Pearson’s chi-square test

*: Fisher’s exact test_._

Most of the patients were men (N = 253, 55.2%) and white (N = 276, 60.3%). The median of age was 49 (IQR, 36–58) years old. The median baseline creatinine and eGFR were 1.4 (IQR, 1.1–1.8) and63 (IQR, 50–74) ml/min/1.73m^2^, respectively. The median time between renal transplantation and hospital admission was 12.5 (IQR, 3–35) months.

### Type of donor and calcineurin inhibitor use

All individuals who received a graft from a deceased donor (N = 285, 62.2%) were induced with thymoglobulin. The majority of patients used a calcineurin inhibitor (N = 416, 90.8%), especially tacrolimus (N = 363, 79,2%).

### Hospital admission causes

Bacterial infections were the most prevalent cause of hospital admission, mainly urinary tract infections (N = 66,14.4%). CMV infection (N = 93, 20.3%) was the most common viral infection.

### Graft biopsy

Among the patients with undetermined graft dysfunction and submitted to graft biopsy, 57 (12.4%) were diagnosed with acute rejection, of which 45 cases showed cellular rejection and 12 developed antibody-mediated rejection. The other 13 patients (2.8%) presented with diagnosis of acute tubular necrosis or changes suggestive of nephrotoxicity.

### Nephrotoxic drugs use

Regarding the nephrotoxic drugs, 76 (16.6%) received contrast media, 55 (12.0%) used vancomycin, 23 (5.0%) amphotericin B, 16 (3.5%) chemotherapy agents and 13 patients (2.8%) received polymyxin-B. Only 1 individual used an aminoglycoside.

### Risk factors for AKI development

The incidence of patients diagnosed with AKI by the KDIGO criteria was 82.3% (N = 377): 146 (31.9%) were classified as KDIGO stage 1, 134 (29.3%) as stage 2 and 97 (21.2%) as stage 3. In a univariate analysis, we identified the following variables associated with AKI: hospital admission due to infections (p = 0.024) and tumors (p = 0.014); time of transplantation (p = 0.051); use of vancomycin (p = 0.004), amphotericin B(p = 0.097), contrast media (p<0.001), mechanical ventilation (p = 0.003), sedatives (p = 0.005) and vasopressors (p<0.001); ICU need (p<0.001), length of ICU stay (p<0.001), baseline creatinine (p = 0.007) and eGFR (p = 0.082)Infection, contrast media use and ICU admission, a marker of severe disease, were the independent risks for AKI. The independent risk factors for AKI development are showed in Table 2.

**Table 2:** Risk factors for acute kidney injury: multivariate analysis.

Variables		OR	95% CI		
			Lower limit	Upper limit	P value
Admission causes	Surgical	reference	reference	reference	Reference
	Infection	5.73	2.61	12.56	<0.001
	CVD	1.00	0.25	3.95	0.990
	Neoplasia	1.08	0.33	3.51	0.894
Contrast media use	No	reference	reference	reference	reference
	Yes	9.34	2.04	42.70	0.004
ICU admission	No	reference	reference	reference	reference
	Yes	8.90	1.77	44.56	0,008

OR:odds ratio, CI: confidence interval, CVD: cardiovascular disease, ICU: intensive care unit

### Risk factors for severe AKI development (in-hospital dialysis need)

Fifty-nine patients (12.8%) required dialysis during their hospital stay.In a univariate analysis the following variables were significant: infection (p<0.001) and rejection (p<0.001); use of vancomycin (p<0.001), amphotericin B(p<0.001), contrast media (p<0.001), vasopressor (p<0.001), mechanical ventilation (p<0.001) and sedatives (p<0.001); time of transplantation (p = 0.002) and length of ICU stay (p<0.001); baseline creatinine (p<0.001) and eGFR (p<0.001).Lower basal eGFR (previous renal dysfunction), need for vasopressor and mechanical ventilation (higher disease severity), tumors and Vancomycin use (nephrotoxic drug exposure) were the independent risks for more severe form of AKI.Regarding the need for in-hospital dialysis, the independent variables are described in the Table 3.

**Table 3:** Risk factors for severe AKI (use of renal replacement therapy): multivariate analysis.

Variables		95% CI				
		OR		Lower limit	Upper limit	P value
Admission causes	Surgical	reference		reference	reference	reference
	Infection	6.41		0.47	87.60	0.164
	Graft dysfunction	207.29		12.37	3474.80	<0.001
	Neoplasia	71.85		3.14	1641.93	0.007
Vancomycin	No	reference		reference	reference	reference
	Yes	3.48		1.06	11.46	0.040
Vasopressor	No	reference		reference	reference	reference
	Yes	9.05		2.41	34.03	0.001
Mechanical ventilation	No	reference	reference		Reference	reference
	Yes	16.37	4.31		62.14	<0.001
eGFR- ml/min		0.96	0.93		0.98	<0.001

AKI: acute kidney injury, OR: odds ratio, CI: confidence interval, eGFR: estimated glomerular filtration rate

### Risk factors for dialysis dependence at hospital discharge

Seventeen patients (3.7%) were dialysis-dependent at discharge. A univariate analysis showed the factors associated with this outcome: infection (p<0.001); rejection (p = 0.048); time of transplantation (p<0.001); baseline creatinine (p<0.001) and eGFR (p<0.001).Acute rejection, transplant time and previous renal dysfunction (higher basal serum creatinine) were the independent risks for more serious sequelae.The independent risk factors for requiring chronic renal replacement therapy are in the Table 4.

**Table 4:** Risk factors for renal replacement therapy at hospital discharge: multivariate analysis.

Variables		95% CI			
		OR	Lower limit	Upper limit	P value
Acute rejection	No	reference	reference	reference	reference
	Yes	5.53	1.31	23.26	0.019
Transplantation time–months		1.03	1.01	1.06	0.003
Creatinine—mg/dl		16.71	5.99	46.64	<0.001

AKI: acute kidney injury, OR: odds ratio, CI: confidence interval, eGFR: estimated glomerular filtration rate

Of the patients who were discharged requiring dialysis, three partially recovered renal function over 12 months of observation: one patient at 2 months of follow-up and the other two patients at 3 months of follow-up. In addition, 2 patients needed to start chronic dialysis: one at 6 months and the other at 11 months after hospital discharge.

### Mortality

Ten individuals (2.1%) died during their hospital stay; all were diagnosed with AKI. We observed, in a univariate analysis, the following factors associated with death: CVD as admission cause (p = 0.009); use of chemotherapy (p = 0.044), vancomycin (p<0.001), amphotericin B(p = 0.001), media contrast (p<0.001), vasopressor (p<0.001), mechanical ventilation (p<0.001) and sedative (p<0.001); ICU admission (p<0.001) and length of ICU stay (p<0.001); in-hospital dialysis need (p<0.001).The independent risk variables for death are showed in the Table 5. There were no other deaths after hospital discharge during the follow-up.

**Table 5:** Risk factors associated with death: multivariate analysis.

Variables		95%CI			
		OR	Lower limit	Upper limit	P value
Admission for CVD	No	reference	reference	reference	reference
	Yes	21.35	2.32	196.49	0.007
Chemotherapy	No	reference	reference	reference	reference
	Yes	21.63	1.06	441.30	0.046
Sedation	No	reference	reference	reference	reference
	Yes	23.82	2.41	235.35	0.007
Length of stay in ICU—days		1.32	1.10	1.59	0.003
In-hospital dialysis		28.41	2.45	329.58	0.007

OR: odds ratio, CI: confidence interval, CVD: cardiovascular disease, ICU: intensive care unit.

### Assessment of renal function after hospital discharge

The creatinine evolution indicated renal function recovery over time (Table 6); however, comparing the groups with and without AKI, patients with AKI had a higher percent change in creatinine levels compared with individuals without AKI (12% x -4.3%; p<0.001). This difference remained even after excluding individuals diagnosed with acute rejection (9.1% x -4.3%; p<0.001) (Table 7).

**Table 6:** Creatinine evaluation in patients with and without AKI according to the KDIGO.

Creatinine-mg/dl	No-AKI	N	AKI	N
Baseline	1.25 (1.10–1.60)	81	1.40 (1.10–1.80)	320
Admission	1.40 (1.10–1.60)	81	1.40 (1.20–1.90)	320
Peak	1.52 (1.20–1.90)	81	2.80 (2.00–3.60)	320
Discharge	1.20 (1.07–1.50)	81	1.84 (1.50–2.40)	298
1 month	1.30 (1.00–1.60)	81	1.80 (1.40–2.30)	298
3 months	1.30 (1.10–1.50)	81	1.70 (1.40–2.20)	298
6 months	1.30 (1.10–1.60)	81	1.60 (1.30–2.00)	298
12 months	1.20 (1.10–1.60)	81	1.50 (1.20–2.10)	298
P*	<0.001		<0.001	

Median (IQR, 25-75^th^), KDIGO: kidney disease: improving global outcomes, AKI: acute kidney injury, N: number of patients,

*Comparison between different times according to the non-parametric Friedman test.

**Table 7:** Assessment of percentage change from baseline creatinine values to 12 months in patients with and without AKI according to the KDIGO criteria.

		No-AKI	AKI	P*
Creatinine (percentage	Median	-4.3%	9.1%	<0,001
change)	1° quartile	-11.8%	-6.7%	
	3° quartile	8.3%	41.7%	
	Minimum	-33.3%	-47.6%	
	Maximum	84.2%	242.9%	

AKI: acute kidney injury, KDIGO: kidney disease improving global outcomes, Median (IQR, 25-75^th^),

*Mann-Whitney test.

## Discussion

As in several studies, the leading cause of hospital admission was infection [10]. Urinary tract and lung infections were prevalent among the bacterial infections [11]. The high prevalence of CMV infection in our sample may be because most of our patients had received kidneys from deceased donors who underwent polyclonal antilymphocyte globulin induction therapy (Thymoglobulin®), known as one of the primary risk factors for CMV infection [12,13].

We detected a high incidence of AKI in renal transplant patients admitted to the hospital. These individuals presented with several intrinsic conditions that predisposed them to AKI development. KDIGO criteria combine the RIFLE (Risk, Injury, Failure, Loss, and End-stage kidney disease) and AKIN (Acute Kidney Injury Network) criteria and enhance the diagnostic sensitivity for AKI.

The exclusion of any pre-renal component as a coadjutant factor for AKI in our patients was not possible. These patients usually undergo multiple interventions, such as hydration, antibiotics, vasopressors, diuretics, and others, hindering analysis of the magnitude of each factor involved in injury. It is important to note that the largest contingent of our patients was diagnosed with more severe AKI (stage 2 or 3).A study reported that the individuals with transient increases in creatinine and renal function recovery within three days after admission presented a 2.26-fold higher risk for death than those without AKI [14]. Therefore, transient changes in renal function also determine unfavorable outcomes.

Infection as a cause of hospitalization was an independent risk factor for AKI development. Most of the current studies report infection as one of the leading causes of AKI [15]. Nakamura *et al*., who assessed only renal transplant patients, found bacterial infection as the primary cause of AKI. It is known that the more severe the infection, the higher the risk is of developing AKI [16].

The use of iodinated contrast was another risk factor for AKI in our sample. Epidemiological studies report the use of contrast media as one of the prevalent causes of AKI in patients. Diabetes and previous renal function impairment are the main risk factors for contrast-induced nephrotoxicity [17]. To our knowledge, there is no specific preventive protocol for renal transplant recipients. Our data indicate the necessity of developing epidemiological studies and specific preventive measures for contrast-induced nephrotoxicity in this population.

AKI usually develops in older patients with sepsis, patients who undergo invasive procedures, and subjects admitted to ICU [18]. In our study, the individuals who required intensive care had a higher risk of an AKI diagnosis, evidencing the importance of closely monitoring patients admitted to these specialized care units.

Despite having a different physiopathology than the AKI observed in other conditions, such as in the context of infections [19], during nephrotoxic drug use [20], post-surgery or during circulatory shock [21], acute rejection is a major cause of renal dysfunction in kidney transplant patients and has a negative impact on graft function in the short and long term [22]. In our study, graft dysfunction, the majority with rejection, was the second leading cause of hospitalization.

We observed that graft dysfunction (most with rejection) and tumors were risk factors for the development of a more severe AKI (i.e., when dialysis was required). AKI is a common complication in patients with cancer and during cancer treatment. It derives from several components, such as preexistent comorbidities, dehydration, nephrotoxicity, sepsis, renal infiltration, glomerulonephritis, and thrombotic microangiopathy [23]. A study with cancer in patients reported a higher incidence of AKI compared with cancer-free patients. Approximately 10% and 4% of patients required nephrology assessment and dialysis, respectively [24].

Individuals with more severe AKI usually develop dysfunction of multiple organs and systems [25], and our data support this finding. Patients who developed hemodynamic instability and respiratory insufficiency and who used vasopressors and underwent mechanical ventilation required dialysis more often. Baseline renal function (lower eGFR) was a risk factor for requiring dialysis, indicating that chronic renal injury is also a major predisposing factor for more severe AKI development.

Vancomycin nephrotoxicity occurs in 10 to 40% of cases [26]. In our study, the use of vancomycin was an independent risk factor for dialysis requirement. The pharmacokinetics of vancomycin is likely altered in renal transplant patients due to the decreased GFR and the presence of multiple drug interactions. Further studies are necessary to assess the actual impact of this drug on renal function in renal transplant patients, and until our findings are confirmed, we suggest that these patients should be treated with other antibiotics with the same efficacy spectrum as vancomycin that are free of nephrotoxic effects.

Current studies in ICU suggest an increase in the number of patients with long-term dependence on dialysis after an AKI event [27]. Our data show that among those patients who required dialysis and survived, one-third of them remained dialysis-dependent after hospital discharge. We observed that the longer the period between renal transplantation and the AKI event, the higher the chance was of the individual being discharged still dependent on dialysis. This observation is most likely due to the loss of functional reserve over time, caused by subclinical alterations such as interstitial fibrosis and glomerular sclerosis.

The baseline renal function and the presence of rejection were also associated with dialysis dependency, again emphasizing the effect of the baseline eGFR in individuals with AKI and the importance of early diagnosis and treatment of acute rejection events to minimize the impact of these events on renal graft function.

The analysis of creatinine measures over time indicated renal function recovery, however, at the end of twelve months of follow-up, the patients with AKI exhibited higher percentage changes in creatinine relative to baseline levels compared with those who did not present with AKI. More importantly, this finding was still observed even after the exclusion of individuals with acute rejection. This phenomenon is also presented in the general population when partial renal function recovery occurs after an AKI event, thus reiterating AKI as both a factor of progression to and a cause of CKD [28].

The main risk factor for progression to chronic kidney disease is pre-existing CKD, especially when associated with proteinuria. Unfortunately we have no data about proteinuria of patients, thus limiting the analysis and the importance of this factor in our sample. It is believed that the renal function loss over time after an episode of AKI is due to a maladaptive repair process. This seems to be due to a persistent inflammatory and pro-fibrotic state. There was an increase of myofibroblasts, extracellular matrix deposition and peritubular-capillaries rarefaction resulting in scarring and renal function impairment [29].

In our study, AKI was not associated with death. The overall mortality rate was very low, and no deaths occurred in the group without AKI, hindering the analysis of this variableas well the risk factors associated with death. This is one of the limitations of our study. Some studies have reported that individuals with CKD who develop certain clinical complications and in whom preexisting CKD worsens exhibit lower mortality rates than those with ‘pure’ AKI [30]. It is possible that renal transplant patients behave alike (i.e., they exhibit high susceptibilities to AKI and a lower mortality).

Probably, patients with CKD and kidney transplant recipients are referenced to nephrologists and conduct tests more frequently allowing the detection of changes in renal function early. On the other hand, patients with AKI have nine times higher risk of developing CKD and twice higher risk of premature death compared to matched controls without AKI and even then, only 60% were seen by a physician and between 10 and 15% were evaluated by a nephrologist in a period of 1 year after AKI episode [31].

Using the new KDIGO diagnosis and classification system, we demonstrated that the incidence of AKI was high and a concern. AKI determined renal function loss at 1-year after hospital discharge. Further studies including a larger number of individuals are necessary to elucidate the impact of AKI on the renal transplant evolution and its outcomes.

## Supporting Information

S1 Table (DOCX)
