# Supplementary material for: Hospital Admission following Acute Kidney Injury in Kidney Transplant Recipients Is Associated with a Negative Impact on Graft Function after 1-Year
Source: PLoS One. 2015 Sep 29;10(9):e0138944. doi: 10.1371/journal.pone.0138944 (PMC4587953; doi:10.1371/journal.pone.0138944)
Supplement: S1 Table — (DOCX) [file pone.0138944.s001.docx]

**Supplementary material**

**S1 Table. KDIGO criteria for acute kidney injury.**

| **Stage** | **Serum Creatinine** |
| --- | --- |
| 1 | 1.5-1.9 times baseline  or  ≥0.3 mg/dL increase |
| 2 | 2-2.9 times baseline |
| 3 | 3 times baseline  or  Increase in serum creatinine to ≥4 mg/dL  or  Initiation of renal replacement therapy |
